# Microeukaryotic Community and Oxygen Response in Rice Field Soil Revealed Using a Combined rRNA-Gene and rRNA-Based Approach

**DOI:** 10.1264/jsme2.ME13128

**Published:** 2014-02-07

**Authors:** Jun Murase, Yuriko Takenouchi, Kazufumi Iwasaki, Makoto Kimura

**Affiliations:** 1 Graduate School of Bioagricultural Science, Nagoya University, Chikusa, Nagoya, 464–8601 Japan; 2 School of Agricultural Science, Nagoya University, Chikusa, Nagoya, 464–8601 Japan

**Keywords:** DGGE, microeukaryotes, rice field soil, protists, rRNA

## Abstract

Irrigated rice field soil is subjected to frequent changes in oxygen status due to the water regime by agricultural management. In this study, the community response of microeukaryotes in rice field soil to the oxygen status was explored in a microcosm experiment under defined conditions. Water-saturated soil was incubated under a two-level factorial design of oxygen and organic enrichment with plant residue. The eukaryotic microbial community composition, which was either present or potentially active in the soils, was analyzed using denaturing gradient gel electrophoresis (DGGE) targeting the 18S rRNA gene or reverse-transcribed 18S rRNA. Oxygen availability was a primary factor shaping the microeukaryotic community in both DNA- and RNA-based analyses, revealing a shift within a week of incubation. Plant residue also affected the microeukaryotic community, which was more notable in the active community showing rRNA expression with time. Sequences of amplicons in DGGE bands indicated that protozoa (ciliates, flagellates, and amoebae) were the most prominent microeukaryotes in water-saturated rice field soil both in DNA- and RNA-based analyses. The use of a modified primer for soil protozoa suggested the functional importance of *Heterolobosea* amoeba in rice field soil, particularly in anoxic soil with organic enrichment.

Microbial eukaryotes play various roles in soil as decomposers, predators, and primary producers. However, understanding the diversity and functions of eukaryotes still represents a major challenge in soil microbiology. Recent studies on microeukaryotic communities in aquatic ecosystems using culture-independent molecular approaches have demonstrated the markedly high diversity covering most lineages of the Eukarya (9, 36). In contrast, the community structure of microbial eukaryotes in soil, except for fungi (1), have been much less thoroughly studied using molecular approaches. Available studies, however, have demonstrated the eukaryotes’ high diversity and geographical heterogeneity, which suggests that they are endemic (2, 10, 23, 28).

The rice field is one of the best-studied model ecosystems of wetlands, which show distinct characteristics from other terrestrial environments in terms of biogeochemistry and microbial ecology (5, 19, 21). The ecological significance of microbial eukaryotes in rice field soil have been demonstrated, *e.g.*, fungi as decomposers of plant residues (32, 33, 41) and protozoa for bacterial predators (26, 29, 30). However, the microeukaryotic community and its response to environmental factors in rice field soil have not been well studied (37).

One of the most important controls of microeukaryotes in the environment is the availability of O_2_ (11, 12). Molecular studies in aquatic environments have demonstrated unexpectedly diverse groups of microeukaryotes in the water layer and sediments that are permanently anoxic (*e.g.*, 6, 18, 39, 40, 42), which indicated an adaptation of the microeukaryotic community to anoxia. Irrigated rice field soil experiences repeated changes in oxygen status due to the agricultural water management of the field, and the microbial eukaryotic community may adapt to this adverse oxygen status. For example, when rice field soil is drained, fungi dominate among the microbial eukaryotes inhabiting the rice straw, and when the rice field is flooded, protozoa are predominant (41). However, the oxygen response of the microeukaryotic community in soil has not been directly addressed; in the field, the change in oxygen status in soil is caused by the change in water potential (flooded vs. drained), which may also affect the diversity and activity of soil microorganisms.

In this study, we explored the oxygen response of the microeukaryotic community of a rice field soil in a microcosm incubation experiment. The community structure of the microbial eukaryotes was analyzed using denaturing gradient gel electrophoresis (DGGE) targeting the 18S rRNA gene and 18S rRNA to understand the oxygen response of the “seed” community and the potentially active community. The universal primer that has been commonly used for molecular analysis of the microeukaryotic community (7) was modified to detect a wide range of soil microeukaryotes, including heteroloboseans, one of the major clades of soil amoeba (38). Our results demonstrated that both present and active microeukaryotes in rice field soil exhibited a rapid response to different (oxic/anoxic) oxygen statuses at the community level with an additional response to organic enrichment with rice straw, which is a major plant residue supplied to the soil.

## Materials and Methods

### Soil

Yellow soil (Oxiaquic Dystrochrepts) was collected from the plough layer (0–15 cm) of the rice field of the Anjo Research and Extension Station, Agricultural Research Center of Aichi Prefecture, Central Japan (34°48′N, 137°30′E) in April, 2008 prior to flooding. The field site and soil properties have been described elsewhere (24). The soil was passed through a sieve (<2 mm) and stored at 4°C until further use within one month.

### Soil incubation

The soil was incubated under oxic or anoxic conditions in a microcosm experiment. For incubation under oxic conditions, 15 g soil was evenly spread on a Petri dish (diameter, 9 cm) and water-saturated with sterilized water mimicking a thin oxic layer (*ca.* 2–3 mm) of flooded rice field soil. For incubation under anoxic conditions, 10 g soil was placed in a pressure culture tube (18 mm diameter, 31 mL Sanshin Industrial, Kanagawa, Japan), and the headspace was flushed with N_2_ gas after the soil was saturated with water. Overall, 42 soil microcosms were prepared for each treatment, to half of which was added pulverized rice straw (*Oryza sativa* var. Nipponbare) that was collected after harvesting in 2007 at 6 mg g^−1^ dry soil to study the response of the microeukaryotic community to organic enrichment. The Petri dishes and tubes were incubated at 25°C in the dark for 10 weeks. The Petri dishes with soil were weighed prior to incubation, and the water loss due to evaporation during incubation was compensated for with sterilized water. Triplicate dishes and tubes for each treatment were periodically sacrificed for analysis of Fe(II) as a proxy of a redox indicator and molecular analysis of the eukaryotic community. For molecular analysis, 0.5 g (wet weight) soil was placed in a 2 mL tube with 0.7 g zirconium beads and stored at −80°C until further use.

### Fe(II) determination

To monitor the redox conditions of the incubated soil, 2 g soil was extracted using 25 mL of 1 M sodium acetate buffer (pH 2.8), and Fe(II) levels were determined by a colorimetric method using *o*-phenanthroline (22).

### DNA and RNA extraction

DNA was extracted from 0.5 g (wet weight) soil using a Fast DNA SPIN kit (MP Biomedicals, Solon, OH, USA) according to the manufacturer’s instructions. DNA was eluted from the binding matrix with 100 μL DNase-free water and stored at 4°C.

RNA was extracted from the soil according to Lueders *et al.* (25) with a minor modification: the stored soil sample was treated with RNA *later*-ICE (Life Technologies, Carlsbad, CA, USA) according to the manufacturer’s instructions prior to RNA extraction. RNA was purified on a filtration column (Zymo-Spin IV-HRC Column; Zymo Research, Irvine, CA, USA), and the co-eluted DNA was subsequently digested with DNaseI (Promega, Madison, WI, USA). The RNA was precipitated with ethanol, dissolved in 30 μL TE buffer, and stored at −80°C.

The RNA/DNA mixtures of selected samples that were not treated with DNase were used to compare the effect of different DNA extraction protocols on the results of the molecular analysis of the microeukaryotic community.

### PCR-DGGE and RT-PCR-DGGE

To amplify the 18S rRNA gene within a wider range of the microeukaryotic community than can be covered with the universal primer Euk516r (7), the primer was modified on the basis of comparisons of the 18S rRNA gene sequences in the ARB database. The modified primer (Euk516rJM2) consists of multiple sequences (TGGCACCAGACTTKYCCTC). This primer successfully amplified the 18S rRNA gene of two *Heterolobosea* amoebae and one lobose amoeba, which were isolated from a rice field soil, with several mismatches in the priming site of Euk516r (Supplementary data, Fig. S1a). When the results of RT-PCR-DGGE of soil RNA using the modified and original primers were compared, the modified primer provided additional DGGE fragments with the original banding pattern using Euk516r maintained (Supplementary data, Fig. S1b). Sequences of the additional DGGE fragments were affiliated with *Naegleria*, a *Heterolobosea* amoeba that is common in soil and lobose amoeba (Supplementary data, Fig. S1), which demonstrates the usefulness of the modified primer in covering a wide range of soil microeukaryotes.

The PCR reaction mixture (50 μL) contained 1 μL DNA sample, 25 pmol of each primer, 200 μM of each deoxyribonucleoside triphosphate, 2.5 U *ExTaq* polymerase (TaKaRa, Otsu, Japan), and 0.1 volume of the 10× PCR buffer provided with the enzyme. The forward primer was Euk1A (7) and Euk516rJM2 was attached with a GC clamp (Euk516rJM2-GC) (7). The PCR program included an initial denaturation step of 130 s at 94°C, followed by 35 cycles of denaturation (30 s, 94°C), primer annealing (45 s, 57.5°C), and primer extension (130 s, 72°C), and a final extension step of 7 min at 72°C. The 18S rRNA gene from the soil samples was amplified using 1 μL DNA samples under these conditions.

The 18S rRNA was amplified from extracted RNA using reverse transcription (RT) followed by PCR of the cDNA according to Murase and Frenzel (29) with some modifications: 10 pmol each of primers Euk1A and Euk516rJM2-GC was added to a 50 μL reaction mixture. The primer annealing temperature was 57.5°C. Complete removal of DNA from RNA samples was confirmed using PCR in the absence of the reverse transcriptase.

Amplicons of the 18S rRNA gene were analyzed using DGGE (denaturing gradient: 20–50%; 100% denaturant contained 7 M urea and 40% [v/v] formamide), and DGGE bands were excised and subjected to sequencing as previously described (31). For DGGE of the amplicons of the reverse transcribed 18S rRNA, the denaturing gradient was established at 25–45%, which better resolved the banding patterns.

### Statistical analysis

Cluster analysis of DGGE patterns was performed using PRIMER-E software (Plymouth Marine Laboratory, Plymouth, United Kingdom). Binary data (presence or absence) of DGGE bands were used to calculate the Bray-Curtis similarity. ANOSIM2 analysis was used to test the null hypothesis that there was no effect of the treatments on the DGGE profiles. Pairwise comparisons were performed by obtaining the DGGE profiles of the same treatment and analyzing them as one group (4).

### Sequencing amplicons in the DGGE bands

DGGE fragments were excised from the gels. After two rounds of re-amplification and confirming the mobility of the amplicons on a DGGE gel, the amplicons were sequenced either directly or after cloning as previously described (32).

### Nucleotide sequence accession numbers

The sequences are available in the DDBJ/EMBL/GenBank nucleotide sequence databases under accession numbers AB769302– AB769376.

## Results

### Fe(II) in soil

**Fig. 1:**
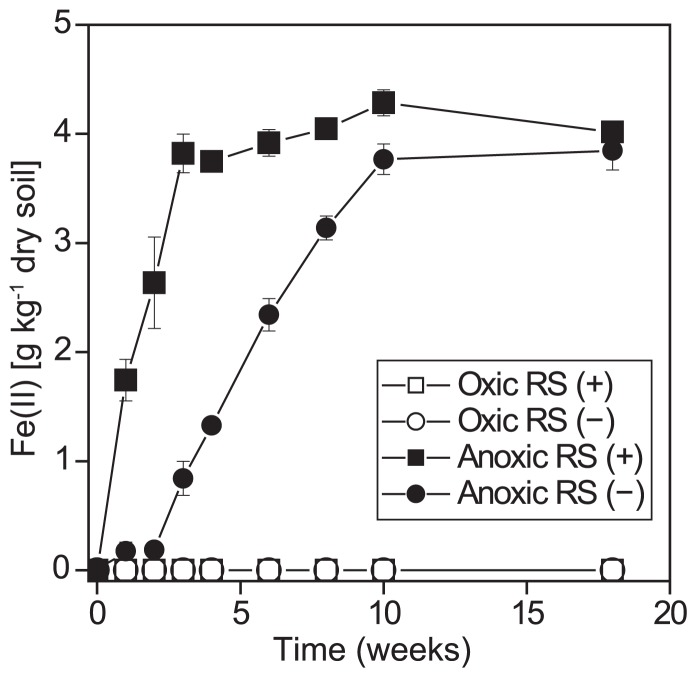
Fe(II) concentration in soil incubated under anoxic or oxic conditions with (+) or without (−) rice straw (RS). The values are expressed as the mean ± standard error (*n*=3).

The concentration of Fe(II) in soil increased with time when the soil samples were incubated under anoxic conditions, which indicated that anaerobic metabolism had occurred (Fig. 1). Fe(II) in anoxic soil without rice straw reached a plateau in 10 weeks. The addition of rice straw to the soil stimulated Fe reduction, and the Fe(II) concentration reached a plateau in 4 weeks. In contrast, no detectable Fe(II) accumulated in soil incubated under oxic conditions with or without rice straw.

### DNA- and RNA-based DGGE of microeukaryotic community

The DNA-based DGGE banding patterns of the soil microeukaryotic community were distinct from the RNA-based DGGE banding patterns in all four different soil incubations (Supplementary data, Fig. S2). The DGGE profiles of amplified DNA extracted independently or together with RNA were nearly identical, which indicated that the DNA extraction procedures did not affect the results of DGGE. We also tested random hexamers as primers in the reverse transcription of 18S rRNA and found that the DGGE profiles were close to those obtained using the PCR reverse primer for reverse transcription (data not shown).

**Fig. 2:**
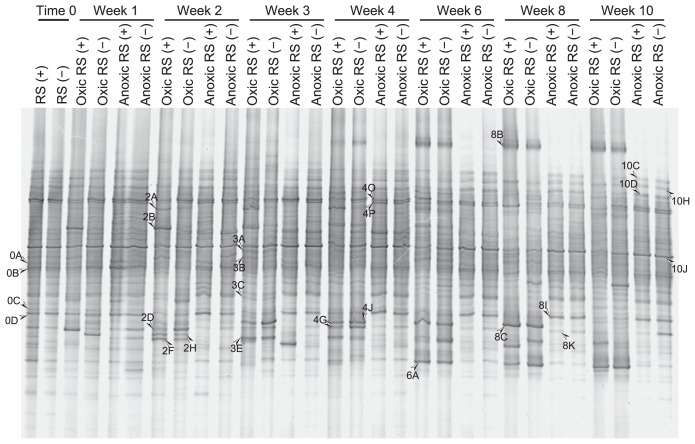
DGGE banding patterns of the amplicons of the 18S rRNA gene over time from soil incubated with (+) or without (−) rice straw (RS) under oxic or anoxic conditions.

We amplified the 18S rRNA genes of microeukaryotes in the soil at the start of incubation and then weekly or biweekly for 10 weeks (a representative DGGE gel is shown in Fig. 2; see Supplemental Fig. S3 for DGGE profiles of all triplicate samples. DGGE bands detected in common in triplicate were regarded as representatives.). We detected 44 bands of different mobility. Each sample had 16–24 bands, 7 of which were commonly observed in all samples. The soils incubated under oxic conditions yielded some additional bands with time, and most of these additional bands were detected in amplified DNA from soils with and without added rice straw. The soil incubated under anoxic conditions yielded a relatively stable DGGE banding pattern. Cluster analysis of the DGGE profiles demonstrated distinct differences in the microeukaryotic community in soil incubated under oxic and anoxic conditions after one week, although the average similarity was high (approximately 80%) (Fig. 4A). Clustering also indicated that the DGGE profiles of amplified DNA from oxic soils shifted with the time of incubation, but rice straw in the soil had little influence. The DGGE profiles of the amplified DNA from anoxic soil also shifted temporally, but this shift was much weaker compared to the shifts in oxic soil, and rice straw had little effect on these profiles.

**Fig. 3:**
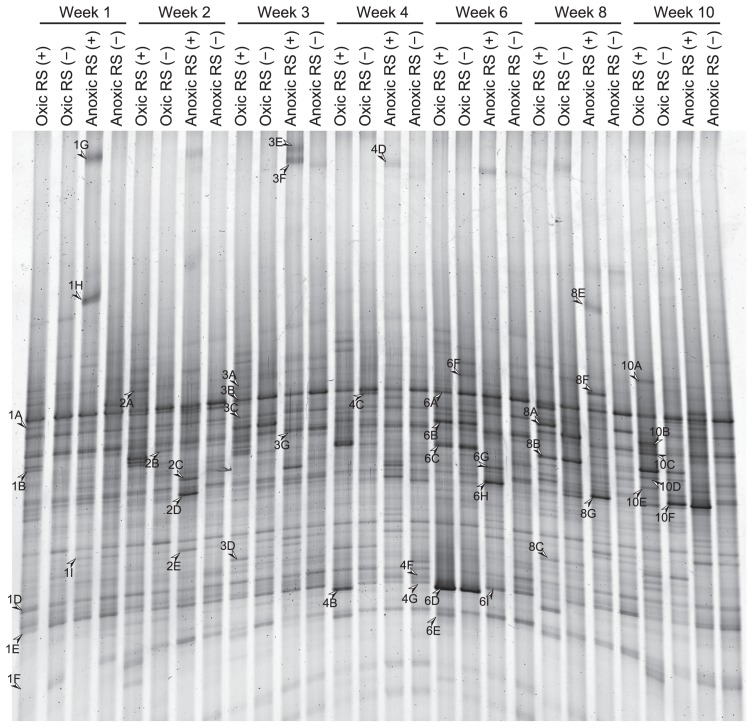
DGGE banding patterns of the amplicons of reverse-transcribed 18S rRNA over time from soil incubated with (+) or without (−) rice straw (RS) under oxic or anoxic conditions.

**Fig. 4:**
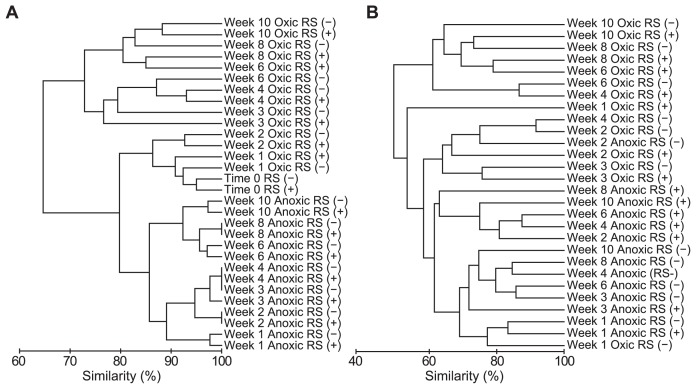
Cluster analysis of the microeukaryotic community based on the DGGE banding patterns of (A) amplicons of the 18S rRNA gene and (B) amplicons of reverse-transcribed 18S rRNA.

The RNA-based DGGE banding patterns were relatively simple compared to those of DNA-based DGGE (Fig. 3; see Supplemental Fig. S4 for DGGE profiles all of the triplicate samples). We detected 56 bands of different mobility. Each sample had 10–22 bands, and 5 bands were commonly observed in all of the samples throughout and before incubation (Supplementary Fig. S4). The effect of oxygen status and rice straw on the microeukaryotic community was more pronounced in RNA-based analysis than DNA-based analysis, which was clearly shown by less similarity between treatments in cluster analysis (Fig. 4B).

An ANOSIM2 test indicated that the effect of the treatment (oxic/anoxic; with/without rice straw) on both DNA- and RNA-based DGGE profiles was significant (P<0.001). In DNA-based analysis, pairwise R-values between soil with or without added rice straw were lower than those of other comparisons (Table 1). The effect of rice straw on DGGE profiles evaluated by pairwise R-values was the most prominent in RNA-based analysis of anoxic soils.

### Phylogenetic affiliation of sequences of the amplicons in DGGE bands

Amplicon sequences in the bands commonly observed in DNA-based analysis were affiliated with microalgae, amoebae, and flagellates (Table 2). Sequences of DGGE fragments exclusively observed under oxic conditions were affiliated with amoebae, *Rhodophyta*, and *Metazoa*, including *Ostracoda*, *Nematoda*, and *Gastricha*. Among these eukaryotes, the bands that were prominent in the late period of incubation in oxic soils (bands 8B and 6A, Fig. 2) represented lobose amoebae and nematodes. Moreover, a few bands were transiently dominant in oxic soils with added rice straw in the early period of incubation (bands 2A and 2B), representing lobose amoebae. DNA sequences in prominent bands from soils incubated under anoxic conditions were affiliated with *Polymyxa* (band 8I) and heterolobose amoebae (band 8K).

Additional sequences were retrieved from DGGE fragments in RNA-based analysis, which were classified on the basis of their appearance on DGGE gel in response to oxygen status, organic enrichment, and incubation time (Table 3). Sequences of DGGE fragments detected in all of the samples were affiliated with fungi, ciliates, flagellates, and amoebae, including *Heterolobosea*. Sequences of DGGE fragments common in oxic soils included those of ciliates, flagellates, amoebae, and polychaetes. Sequences of DGGE fragments from oxic soils with rice straw represented various phylogenetic groups, including dominant groups of fungi, ciliates, and amoebae. Sequences of DNA in bands of oxic soil samples without rice straw were mainly affiliated with ciliates, amoebae, and fungi belonging to *Chytridiomycota*. Sequences of DGGE fragments from anoxic soil samples with added rice straw were closely affiliated with anaerobic ciliates (*Metopus*), anaerobic/microaerophilic heteroloboseans (*Harpagon*), and oomycetes. Sequences affiliated with testate amoebae and *Polymyxa* were obtained from the bands of anoxic soil samples with and without added rice straw.

## Discussion

In this study, we saturated rice field soil with water for both oxic and anoxic incubation to analytically study the response of microbial eukaryotes to oxygen while avoiding the effect of soil moisture, which would also affect the community composition and activity of soil microorganisms (27, 43). Analysis of Fe(II) validated that oxic and anoxic conditions were maintained in each incubation, although the oxygen level of water-saturated soil in the oxic incubation may have been lower than in unsaturated soil under upland conditions. Soils incubated under anoxic conditions with added rice straw rapidly accumulated Fe(II) and reached a plateau in 4 weeks, which suggested that organic enrichment with rice straw stimulated anaerobic respiration, giving further reduced conditions after 4 weeks, where methanogenesis was the terminal process (20).

Both DNA- and RNA-based analyses clearly demonstrated that oxygen status shaped the community structure of microbial eukaryotes both in the present and active forms in the soil. DGGE bands of amplicons specific either to aerobic soil or anaerobic soil were identified. These results suggested that microbial eukaryotes with different physiologies in response to oxygen inhabited the rice field soil and the community adapted to the given oxygen status, which developed within a short period of time (<1 week). The rapid response of the bacterial community in rice field soil to the oxygen gradient after flooding has also been previously reported (34).

Although less influential than the oxygen status, the response of the microeukaryotic community to organic enrichment was also manifested using cluster analysis, particularly for RNA-based DGGE. Various functional groups of microeukaryotes, such as decomposers (fungi) and bacterial predators (protozoa), expressed the 18S rRNA gene in response to the addition of rice straw. This demonstrated the sensitivity of the RNA-based approach in studying the effect of different environmental factors on functionally important microbial eukaryotes in soil. It is generally assumed that RNA-based analysis targets the population of microorganisms that are actively growing in these environments. However, this is not always true (3); specific levels of 18S rRNA are maintained in the resting form of microbial eukaryotes, such as protozoan cysts (44) and fungal spores (15). Indeed, some major DGGE bands in RNA-based analysis that were included in all of the samples (*e.g.*, band 3C and 6A in Fig. 3, related to ciliate and fungi *Mortierella*, respectively) were also detected in the control soil that had been stored at 4°C for more than one year, which indicated they were most likely in dormancy. In this study, we regarded DGGE bands in RNA-based analysis that appeared in samples during incubation as representing active forms of microbial eukaryotes.

Combined DNA- and RNA-based community analyses demonstrated a distinct difference between the seed bank and active community of microbial eukaryotes in rice field soil. For example, one prominent DGGE fragment exclusively observed in rRNA-gene-based analysis (band 10J in Fig. 2) demonstrated a close relationship with planktonic green alga *Desmodesumus* (formerly *Scenedesmus*), which is commonly observed in rice fields (14). This suggested that rice field soil stores not only the seeds of soil microeukaryotes but also that of aquatic microeukaryotes that proliferate when the soil is flooded for rice cultivation.

Most amplicon sequences in DGGE bands, particularly those from RNA-based analysis, were affiliated with different types of protozoa (ciliates, flagellates, lobosea amoebae, heteroloboseans), while only a few of these sequences were affiliated with fungi. These results were in contrast with our previous results that fungi are actively involved in the decomposition of plant residue in the same rice field soil that was unsaturated with water (33, 41). Such differences suggest that water potential is another environmental factor that potentially shapes the active community of microeukaryotes in rice field soil and that protozoa are functionally more important in water-saturated rice field soil. This is plausible given that soil protozoa inhabit the water film of soil particles (16). The fungal community in rice field soil may also respond to water conditions; in this study, all fungal-related sequences from oxic soil with added rice straw were affiliated with flagellated fungi (*Chytridiomycota*), which are characterized by zoospores and are commonly observed in aquatic ecosystems as well as in soil (17). In contrast, the fungal community in aerated rice field soil is often dominated by other groups such as *Ascomycota*, *Mucoromycotina*, and *Zoopagomycotina* (32). Further studies are required to elucidate the effect of soil moisture conditions on the microeukaryotic community structure in rice field soil.

Ciliate-related sequences were not identified in amplicons in prominent bands of DNA-based DGGE, while some were identified in amplicons in prominent bands of RNA-based DGGE (*e.g.*, band 6B and 8F in Fig. 4). In many soils, ciliates are less numerous than flagellates and amoebae (8); however, our results suggested that the activity of ciliates might be comparable to that of other protozoa in terms of rRNA expression. Because different sequences related to ciliates were identified in samples incubated for different periods and with different treatments, ciliates could potentially be used as bioindicators in rice field soil to show the impact of agricultural practices such as soil and water management, fertilization, and pest control on soil microorganisms (13).

Our RNA-based analysis revealed the involvement of anaerobic protozoa in response to the addition of rice straw. In particular, the detection of sequences related to *Harpagon* confirms the utility of the modified primer adapted to amplify *Heterolobosea* 18S rRNA, which was not amplified with the original primer (Euk 516r), and also suggests the stimulated activity of this group in anaerobic rice field soils with organic enrichment. *Harpagon* is an amoebo-flagellate and a member of the *Psalteriomonadidae*, which consists of anaerobic/microaerophilic heterolobosea (35). The *Harpagon* species are often isolated from freshwater sediments and feed on bacterial prey. This species may play an important role as a grazer in anaerobic rice straw decomposition because the anaerobic microbial food chain must be much shorter than the aerobic chain, due to low growth efficiency (11). Importantly, no cysts have been reported for this family (35). Thus, it would be interesting to study their survival strategy in rice field soil under aerobic conditions.

## Conclusion

The combined DNA/RNA-based approach revealed a distinct difference between the seed bank and the active portion of microbial eukaryotes in rice field soil. Both present and active communities of microeukaryotes demonstrated a rapid response to the oxygen status, resulting in a shift within one week. The use of a primer modified for soil protozoa suggested the ecological importance of heteroloboseans, particularly in the anoxic decomposition of plant materials. In this study, we adopted two contrasting conditions in oxygen status, while actual wetland soil consists of layers of different oxygen contents. Future studies will focus on the effect on the oxygen gradient on microbial eukaryotes in soil. The ecological functions of anaerobic protozoa in rice field soil should also be addressed further.

## Supplementary data



## Figures and Tables

**Table 1:** Pairwise R values of ANOSIM test*

	Oxic RS (+)	Oxic RS (−)	Anoxic RS (+)	Anoxic RS (−)
Oxic RS (+)		−0.031	0.693	0.695
Oxic RS (−)	0.104		0.581	0.599
Anoxic RS (+)	0.726	0.658		−0.126
Anoxic RS (−)	0.796	0.597	0.566	

* Above diagonal: rRNA-gene-based DGGE (global R = 0.405), below diagonal: rRNA-based DGGE (global R = 0.565); RS, rice straw.

**Table 2:** Similarities of sequences obtained from the excised bands of rRNA-gene-based DGGE to sequences in the NCBI database

Band		Appearance^1^	Seq. bp	Closest relative		Similarity (%)	Phylogenetic group

				Microorganism	Accession number		
0	A	Common	444	*Flamella arnhemensis*	EU186022	83	Amoebae, Lobosea
0	B	Common	527	*Scotiellopsis terrestris*	AB012847	100	Green algae, Chlorophyta
0	C	Common	517	*Diplophrys marina*	AF265331.	80	Stramenopiles
0	D	Common	525	*Arachnula* sp. ATCC 50593	EU273440	86	Flagellates, Rhizaria
4	O	Common	531	Soil flagellate AND21	AY965866	97	Flagellates, Rhizaria
4	P	Common	539	Lobosea species Mb_5C	AB425950	97	Amoebae, Lobosea
10	J	Common	405	*Desmodesmus communis*	X73994	98	Green algae, Chlorophyta
10	C	Common	524	*Ripidomyxa* sp. RP009	AY549563	79	Amoebae, Lobosea
10	D	Common	527	Stramenopile species MAST-12 KKTS_D3	EF219381	97	Stramenopiles
10	H	Common	537	*Ceratiomyxella tahitiensis* HI04-93-l-1	FJ544419	85	Amoebae, Lobosea
2	D	Oxic	523	*Ilyocypris japonica*	AB076616	99	Ostracoda, Metazoa
2	F	Oxic	485	*Cyanidioschyzon* sp. Y16	HM161754	86	Rhodophyta
2	H	Oxic	487	*Cyanidioschyzon* sp. Y16	HM161754	86	Rhodophyta
3	A	Oxic	537	*Nolandella* sp. ATCC PRA-27	EU273456	87	Amoebae, Lobosea
3	B	Oxic	500	*Acanthamoeba polyphaga* ATCC 30872	AY026244	99	Amoebae, Lobosea
3	C	Oxic	554	*Tracheleuglypha dentata*	AJ418790	99	Testate amoeba, Rhizaria
3	E	Oxic	524	*Chaetonotus* sp.	AJ001735	95	Gastrotricha, Metazoa
4	G	Oxic	537	*Ilyocypris japonica*	AB076616	99	Ostracoda, Metazoa
4	J	Oxic	537	*Ilyocypris japonica*	AB076616	99	Ostracoda, Metazoa
6	A	Oxic	525	*Eumonhystera filiformis* EumoFil2 829+830	AY593937	99	Nematoda, Metazoa
8	B	Oxic	596	*Saccamoeba limax*	AF293902	79	Amoebae, Lobosea
8	C	Oxic	536	*Heterocypris incongruens* C3	EU370424	93	Ostracoda, Metazoa
2	A	Oxic RS (+)	460	*Flamella fluviatilis*	EU186024	83	Amoebae, Lobosea
2	B	Oxic RS (+)	441	*Flamella fluviatilis*	EU186024	82	Amoebae, Lobosea
8	I	Anoxic	536	*Polymyxa graminis*	AF310898	99	Plasmodiophorida, Cercozoa
8	K	Anoxic	627	*Naegleria pagei* strain 4830/I	DQ768721	98	Amoebae, Heterolobosea

1 Common, commonly observed; Oxic, prominent in oxic soils; Anoxic, prominent in anoxic soils; Oxic RS (+), prominent in oxic soils with added rice straw

**Table 3:** Similarities of sequences obtained from the amplicons in excised bands of rRNA-based DGGE gels to sequences in the NCBI database

Band	Presence in treatment^1^				Seq. bp	Closest relative		Similarity (%)	Phylogenetic group
	
	Oxic RS (+)	Oxic RS (−)	Anoxic RS (+)	Anoxic RS (−)		Microorganism	Accession number		
1D	0–10	0–10	0–10	0–10	535	*Centropyxis laevigata*	AY848965	99	Amoebae, Lobosea
1E (4G, 6I)	0–10	0–10	0–10	0–10	617 (617, 617)	*Naegleria lovaniensis*	U80062	97 (97, 97)	Amoebae, Heterolobosea
1F	0–10	0–10	0–10	0–10	609	*Naegleria* sp. RR11Z/I	DQ768722	93	Amoebae, Heterolobosea
3B (6A)	0–10	0–10	0–10	0–10	526 (527)	*Mortierella alpina*	AY546097	99 (100)	Fungi, Mortierellomycotina
3C	0–10	0–10	0–10	0–10	427	*Anteholosticha monilata*	GU942567	93	Ciliates, Alevolata
2B	0–3	0–10	0–10	0–10	538	*Protaspis* sp. CC-2009b	FJ824125	87	Flagellates, Cercozoa
3G	1–6	1–6	1–10	1–10	537	*Arachnula* sp. ATCC50593	EU273440	93	Flagellates, Cercozoa
1A	1	1	1		521	*Gastrostyla steinii*	AF508758	95	Ciliates, Alevolata
3D	3–4	3–4			400	*Thraustochytriidae* sp. HU1	DQ367047	79	Stramenopiles
4B (6D)	4–6	4–6			536 (441)	*Aeolosoma hemprichi*	AJ310500	99 (99)	Polychaetes, Metazoa
6B	3–8	3–8			521	*Hypotrichida* sp. LPJ-2005	DQ022066	98	Ciliates, Alevolata
6C (8B)	6–10	6–10			527 (527)	*Arachnula impatiens*	EU567294	95 (96)	Flagellates, Cercozoa
8C	8–10	8–10			436	*Cryptodifflugia operculata*	JF694280	97	Amoebae, Lobosea
10D	8–10	6–10			559	*Tubulinida* sp. MK-2011	HQ687486	86	Amoebae, Lobosea
1B	1–3				530	*Albugo laibachii*	FR832884	97	Oomycetes, Stramenopiles
2A	2				516	*Platyreta germanica*	AY941200	79	Cercozoa
3A	3				526	*Allochytridium expandens*	AF164291	95	Chytridiomycota, Fungi
4A	4				524	*Dioszegia* sp. TY-217	AY313036	84	Basidiomycota, Fungi
6E	4–10				614	*Naegleria pagei* 4830/I	DQ768721	100	Amoebae, Heterolobosea
8A	8				508	*Spizellomyces* sp. NBRC 106283	AB586079	90	Chytridiomycota, Fungi
10A	10				516	*Pseudocyrtolophosis alpestris*	EU264564	99	Ciliates, Alevolata
10B	10				521	*Anteholosticha monilata*	GU942567	98	Ciliates, Alevolata
10C	10				432	*Theratromyxa weberi*	GQ377666	94	Amoebae, Rhizaria
10E	10				531	*Triparticalcar arcticum*	DQ536480	88	Chytridiomycota, Fungi
4C		4			520	*Uroleptus pisces*	AF164131	99	Ciliates, Alevolata
6F		6			584	*Bryometopus pseudochilodon*	EU039887	96	Ciliates, Alevolata
10F		8–10			556	*Tubulinida* sp. MK-2011	HQ687486	81	Amoebae, Lobosea
1I (2E)			1–10	1–10	531 (437)	*Polymyxa betae*	AF310902	97 (97)	Plasmodiophorida, Cercozoa
4F			4	4	537	*Centropyxis laevigata*	AY848965	97	Amoebae, Lobosea
1G (3E)			1–3		509 (509)	*Harpagon descissus*	JN606329	100 (100)	Amoebae, Heterolobosea
1H			1		509	*Harpagon descissus*	JN606329	99	Amoebae, Heterolobosea
2C (6G)			2–10		530 (530)	*Pythiaceae* sp. *PHY2*	FJ794934	99 (99)	Oomycetes, Stramenopiles
2D (6H, 8G)			2–10		530 (530, 530)	*Lagenidium myophilum*	AB284577	99 (99, 99)	Oomycetes, Stramenopiles
3F			3		509	*Harpagon descissus*	JN606329	97	Amoebae, Heterolobosea
4D			4		509	*Harpagon descissus*	JN606329	99	Amoebae, Heterolobosea
8E			8		509	*Harpagon schusteri*	JN606340	99	Amoebae, Heterolobosea
8F			8		494	*Metopus palaeformis*	AY007452	98	Ciliates, Alevolata

1 Incubation time (weeks) when each band was detected.
