# Supplementary material for: Microeukaryotic Community and Oxygen Response in Rice Field Soil Revealed Using a Combined rRNA-Gene and rRNA-Based Approach
Source: Microbes Environ. 2014 Feb 7;29(1):74–81. doi: 10.1264/jsme2.ME13128 (PMC4041227; doi:10.1264/jsme2.ME13128)
Supplement: Supplementary file 1 [file 29_74_s1.pdf]

Supplementary data

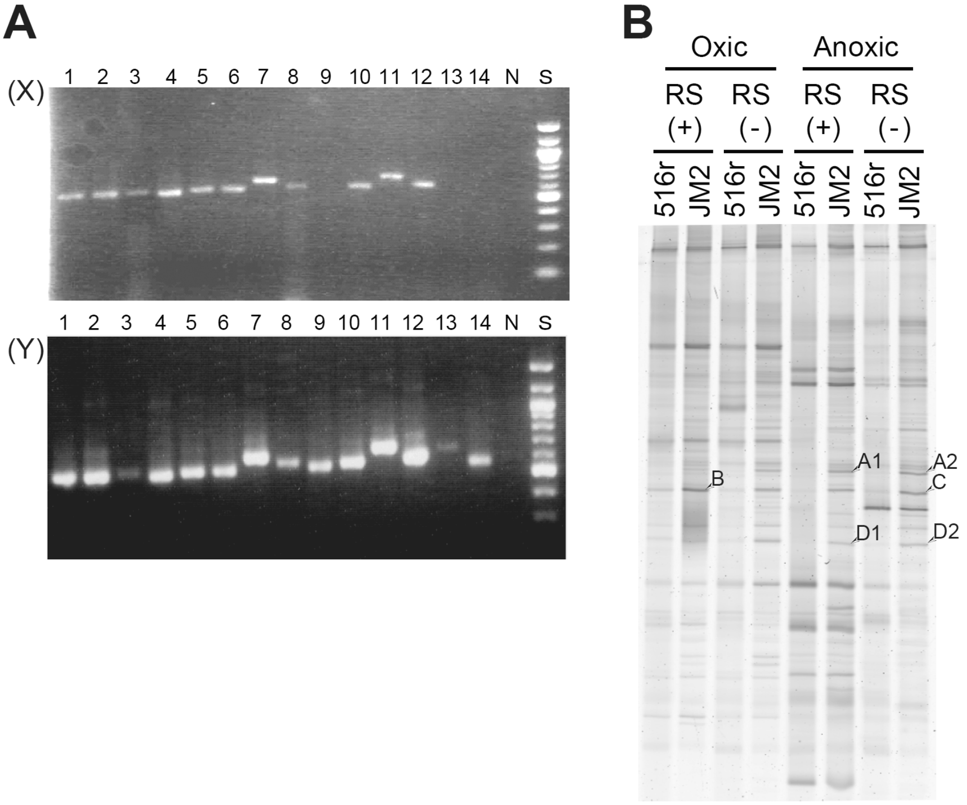

Fig. S1. (A) Amplification of the 18S rRNA gene from protozoan DNA using (X) Euk 1A and 516r and (Y) Euk 1A and 516rJM2. 1, *Colpoda* sp. (0); 2, *Euplotes* sp. (0); 3, *Cercomonas* sp. (0); 4, unidentified flagellate (0); 5, *Spumella* sp. (0); 6, *Adriamonas* sp. (0); 7, *Acanthamoeba* sp. (0); 8, *Hartmannella* sp. (0); 9, *Filamoeba* sp. (3); 10, unidentified lobose amoeba (0); 11, *Platyamoeba* sp. (1); 12, lobose amoeba (0); 13, *Vahlkampfia* sp.(3); 14, *Vrihiamoeba italica* (3). The numbers of mismatches of the sequences to the 516r primer are indicated in parentheses. (B) The effect of modification of the reverse primer on DGGE banding patterns of the amplicons of reverse-transcribed 18S rRNA from soil incubated for 6 weeks with (+) or without (−) rice straw (RS) under oxic or anoxic conditions. 516r, the original primer; JM2, the modified primer. Sequences of bands A, B, and C could be affiliated with *Naegleria*, Heterolobosea amoeba common in soil and that of band D was distantly related to lobose amoeba. Sequences are available in the DDBJ/EMBL/GenBank nucleotide sequence databases under the accession numbers AB781929-AB781934.

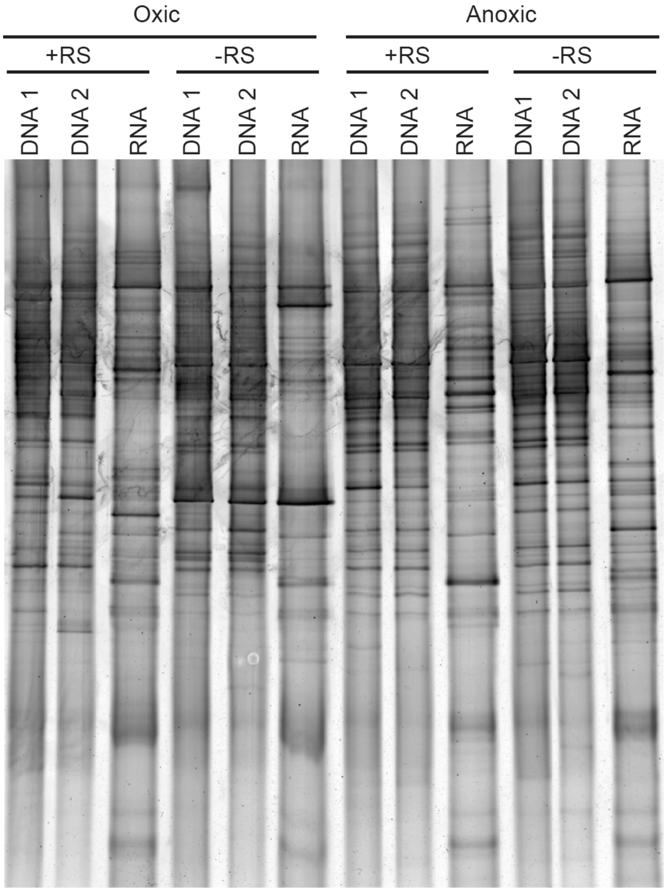

Fig. S2. DGGE banding patterns of amplicons of the 18S rRNA gene and reverse-transcribed 18S rRNA of the microeukaryotic communities in rice field soil incubated under oxic or anoxic conditions with (+) or without (−) rice straw (RS). rRNA and DNA were extracted from soil incubated for 4 weeks. DNA 1, independently extracted DNA; DNA 2, DNA co-extracted with RNA. The denaturing gradient was 20-50%.

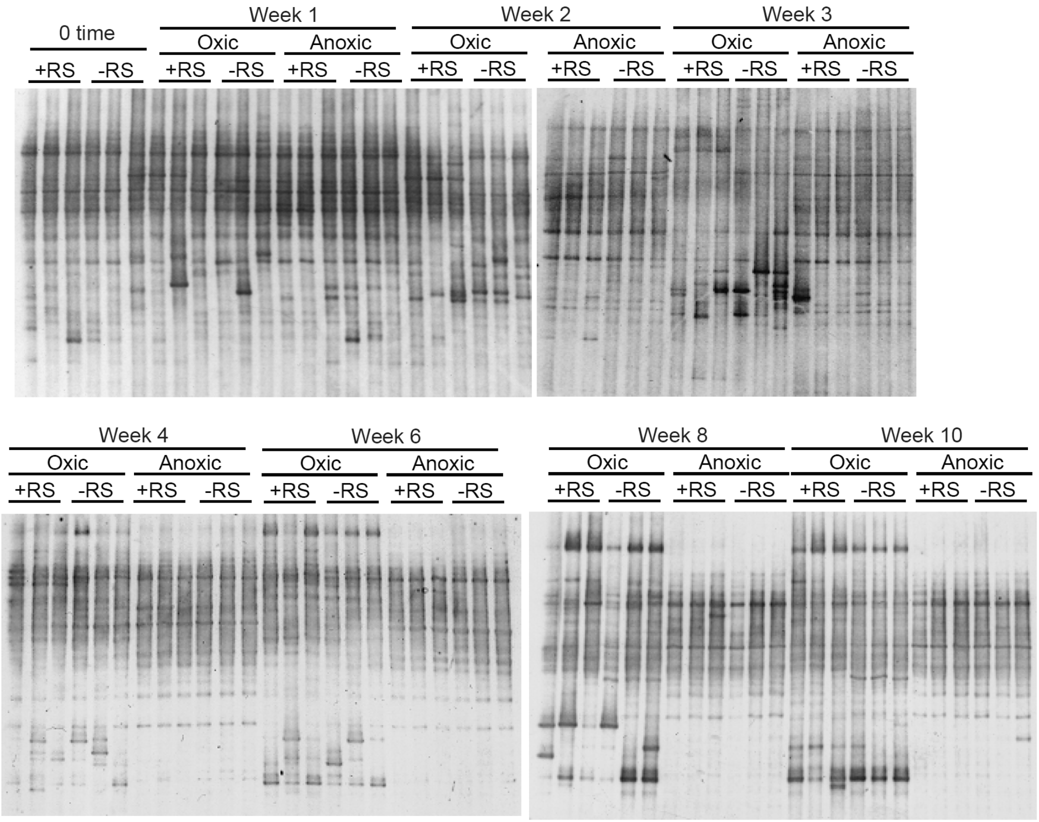

Fig. S3. Comparison of rRNA-gene-based DGGE patterns of three replicates.

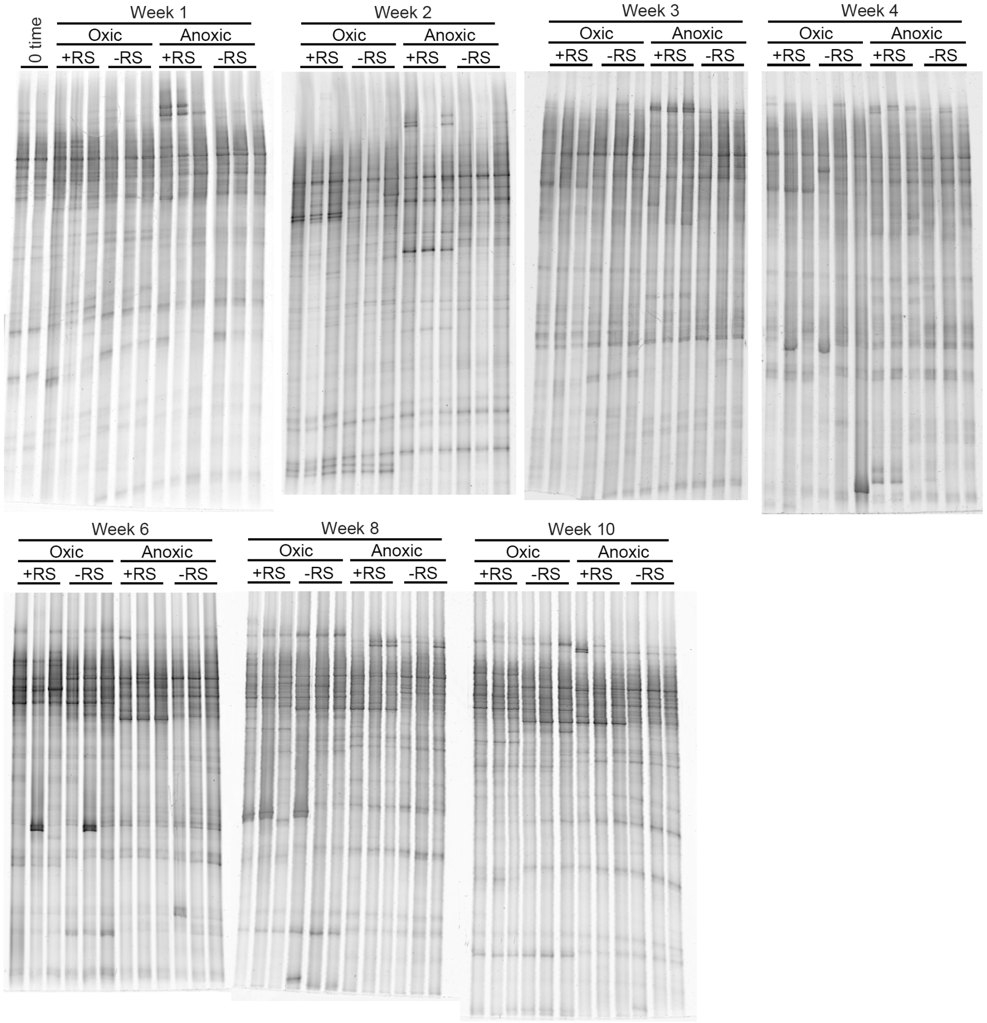

Fig. S4. Comparison of rRNA-based DGGE patterns of three replicates.
